# Neurophysiologic findings in children with spastic cerebral palsy

**DOI:** 10.4103/1817-1745.66671

**Published:** 2010

**Authors:** Ruchi Kothari, Ramji Singh, Smita Singh, Manish Jain, Pradeep Bokariya, Maria Khatoon

**Affiliations:** Department of Physiology, Mahatma Gandhi Institute of Medical Sciences, Sevagram, Wardha, India; 1 Department of Opthalmology, Mahatma Gandhi Institute of Medical Sciences, Sevagram, Wardha, India; 2 Department of Pediatrics, Mahatma Gandhi Institute of Medical Sciences, Sevagram, Wardha, India; 3 Department of Anatomy, Mahatma Gandhi Institute of Medical Sciences, Sevagram, Wardha, India

**Keywords:** Brainstem evoked potential, developmental delay, spastic cerebral palsy, visual evoked potentials

## Abstract

**Context:**

Cerebral palsy (CP) is a heterogeneous group of permanent, non-progressive motor disorders of movement and posture caused by chronic brain injuries. It is the most common cause of physical disability in childhood; spastic cerebral palsy being the most prevalent of its various forms. There is scanty information about the neurophysiologic investigations in children diagnosed as having spastic CP.

**Aims:**

The aim of the study was to investigate the relationship between abnormal VEP and BAEP findings with different clinical parameters in children with spastic cerebral palsy.

**Materials and Methods:**

Fifteen children with spastic CP in the age range 4 months to 10 years participated in this study. Evaluation of VEPs, brainstem evoked potentials (BAEPs) were performed in all study patients as well as 35 healthy children as controls. The study was conducted after obtaining ethics committee approval and informed consent of parents.

**Statistical Analysis Used:**

Significance of difference in the mean values of different parameters in different groups was assessed by Student’s “t” test and the *P* value <0.05 was considered to be significant. All the values were expressed as mean ± 1 Std. Deviation.

**Results:**

A significant difference was found in the VEP latencies and amplitude between the subjects with CP and controls. Striking BAEP abnormalities in CP patients include prolongation of absolute latency of wave V, interpeak latencies of III-V and lowered I-V ratio. Abnormal VEPs and BAEPs in children with bilateral spastic cerebral palsy demonstrated a correlation with the presence of moderate to severe developmental delay.

**Conclusions:**

The differences in VEPs and BAEPs were determined between CP children and healthy children. The abnormalities found are probably linked to the neurological deficits present in cases of cerebral palsy.

## Introduction

Cerebral palsy (CP) is a heterogeneous group of permanent, non-progressive motor disorders of movement and posture caused by chronic brain injuries that originate in the prenatal, perinatal, or postnatal period. It is the most common cause of physical disability in childhood.[1,2]

The four major subtypes of CP are spastic, athetoid, ataxic, and mixed cerebral palsy, with spastic forms being the most common (65%).[3] CP prevalence is increasingly encountered in neonatal clinics since more number of premature infants survives because of better neonatal care made available these days.

Besides motor deficits in cerebral palsy, the other causes of handicap are the associated disabilities, in particular intellectual impairment, epilepsy, speech defects, disorders of vision and hearing, and specific educational and behavioral abnormalities, with the result that the majority of sufferers cannot take their place in normal society[4–6] (Crothers and Paine, 1959; Henderson, 1961; Ingram, 1964a).

In recent years, the neurophysiologic examination of children with CP has been of increasing interest to both pediatricians and vision researchers because it provides new insights into mechanisms of brain damage and repair in CP. However, there are only very few reports on electrophysiological investigations on children having cerebral palsy[7–10] and on Visual Evoked Potentials (VEPs) along with Brainstem Auditory Evoked Potentials (BAEPs) in children with CP in this part of the world.

Hence the present study was undertaken to investigate children with spastic cerebral palsy using VEPs and BAEPs and correlate the electrophysiological findings with clinical features.

## Materials and Methods

The study was conducted in the Neurophysiology Unit of the Department of Physiology. A total of 50 subjects were investigated for the evaluation of VEPs and BAEPs after obtaining ethics committee approval for the present study. Fifteen children with spastic CP in the ages ranging from 4 months to 10 years participated as patient group in this study. A control group of 35 children with the same age range and with no significant birth history or visual anomaly was also tested. The number of controls was two times greater than the study group to increase statistical power of this study. Data of spastic CP children were collected retrospectively regarding patient’s age, pre-, peri- and postnatal events, and history of epilepsy. Clinical eye examinations included observation of fixation, following objects, pupillary reflexes, refraction, and fundus examination.

### Recording procedure for VEP and BAEP

The recording was done using RMS EMG. EP MARK II machine manufactured by RMS Recorders and Medicare System, Chandigarh. The stimulus for VEP recording was a monocular flash from light-emitting diode goggles that were held lightly over the infants’ eyes. Flashes were presented at 0.5 per second. Silver cup electrodes were attached with paste and tape, with impedances below 5 kilo-ohms. As per 10-20 International System of EEG placements, the reference electrode (Fz) was placed 12cm above the nasion, the ground electrode (Cz) at the vertex and the active electrode (Oz) at approximately 2 cm above the inion. A band pass of 1 to 100 Hz was employed, the gain was 10,000, the sweep was 1 sec, and automatic artifact reject routine was employed. For BAEP recording, same surface electrodes were used and placed at the vertex (Cz), both ear lobes (Ai and Ac) and forehead (ground). Monoaural auditory stimulus consisting of rarefaction clicks were delivered through an electrically shielded earphone at a rate of 11.1/sec. contra-lateral ear was masked with pure white noise 40 dB below that of BAEP stimulus. A band pass of 150-3000 Hz was used to filter out undesirable frequencies in the surrounding. Responses to 2000 click presentations were averaged for 10 msec.

The testing procedures were explained to a parent of each subject who volunteered to participate and a consent form was signed.

### Parameters studied

- The absolute latencies of the peaks of positive wave P2 and the negative waves N1 and N2 were recorded. The amplitude of P2 is measured from the peak of N2 to the trough of P2 (N2 – P2). The absolute difference in the components evoked by the right eye and left eye stimulation (inter ocular differences) are also determined.
- BAEP threshold for each ear with absolute latencies of I, III, V waves, interpeak latencies of I-III, III-V, I-V and ratio of V to I amplitude (V:I) were considered from recordings for comparison among the two groups.

## Results

VEPs and BAEPs were recorded in the children with CP and compared with healthy controls. The VEP parameters of all the 15 children with CP are given in Table 1. The lowest latency obtained was 111.9 msec and highest value was 146.9 msec with maximum amplitude of the most prominent positivity as 13.24 *µ*v. The range of variation in interocular difference for latency was 1.2 to 16.6 msec and for amplitude was 0.14 to 8.1 *µ*v.

**Table 1:** Visual evoked potentials parameters of the children with cerebral palsy

Case no.	Name of the subject	Sex	RE P2 latency (msec)	RE P2 amp. (µV)	LE P2 latency (msec)	LE P2 amp. (µV)	Inter-ocular diff. in P2 latency	Inter-ocular diff. in P2 amp (µV)
1.	KVM	F	116.3	6.13	114.4	6.31	1.9	0.18
2.	CAK	M	145.6	5.15	132.5	1.4	13.1	3.75
3.	KVD	M	131.3	4.28	139.4	9.73	8.1	5.45
4.	ARG	M	143.1	7.54	131.3	6.74	11.8	0.8
5	JRK	M	133.5	5.35	118.8	1.20	14.7	4.15
6	ASK	M	138.1	10.84	120.8	2.74	17.3	8.1
7.	BDC	M	139.3	1.34	137.5	1.15	1.8	0.19
8.	RGT	M	120.6	2.37	122.5	3.28	1.9	0.91
9.	ONM	M	130.0	10.20	143.8	7.16	13.8	3.04
10.	SBK	M	121.9	5.45	128.1	4.20	6.2	1.25
11.	MVP	M	111.9	7.48	117.5	8.00	5.6	0.52
12.	TVS	M	130.6	12.61	123.8	13.24	6.8	0.63
13	ASN	F	130.3	0.55	146.9	0.41	16.6	0.14
14	NNM	F	139.4	5.81	130.6	6.13	8.8	0.32
15	NDT	M	120.6	8.26	119.4	8.63	1.2	0.37

The mean latencies and amplitude of Flash VEP records in the patient group and control group have been tabulated in Table 2. It is quite evident from the observations on VEP that there is a peculiar characteristic which is outstandingly seen in the patient group i.e. a marked delay in the latency as well as a reduction in the amplitude when compared with the controls. The means for all VEP parameters of patient group were statistically different from the control group (*P*<0.05).

**Table 2:** Mean latencies and amplitudes of VEP recordings

Groups	RE P2 latency (msec)	LE P2 latency (msec)	Inter-ocular diff. in P2 latency	RE P2 amplitude (µV)	LE P2 amplitude (µV)	Inter-ocular diff. in P2 amplitude (µV)
Patient group (mean± SD) No. = 15	130.17±10.10	128.49±10.05	8.64±5.63	6.29±3.30	5.42 ± 3.63	1.99±2.4
Control group (mean± SD) No. = 35	97.7±5.61	97.67±4.51	2.02±2.31	13.01±3.5	13.18±3.29	1.13±1.44
Unpaired t test	*P*<0.05	*P*<0.05	*P*<0.05	*P*<0.05	*P*<0.05	*P*<0.05

Apart from this, the variations in VEPs records in our study were seen as:

1. Unusual and improper waveform as obtained in Case No.2 having CP with microcephaly, mental retardation with seizure disorder.
2. Monocular delayed latency and reduced amplitude as in Case No.8 having CP with typical febrile seizure and mental retardation.
3. Abnormally delayed latencies and missing components as in:
  - Case no.5 with CP with microcephaly, seizure disorder, mental retardation with global developmental delay with corpus callosal agenesis.
  - Case no.7 with CP with microcephaly, mental retardation with global developmental delay with B/L primary optic atrophy.
4. “W” Pattern or bifid P wave morphology as in:
  - Case no.1 with CP with microcephaly, seizure disorder, delayed milestones, mental retardation and partial corpus callosal agenesis
  - Case no.4 with CP with microcephaly, global developmental delay with seizure disorder
5. Broadened “P wave” was obtained in case no.14 having CP with frontal lobe atrophy.
6. Absent waveform was observed in
  - Case no. 13 with CP with sensorineural hearing loss (SNHL). Its MRI revealed cerebral atrophy in B/L hemispheres.

Out of the total 15 cases, the presenting features of 10 children with spastic CP along with some associated abnormalities such as microcephaly, mental retardation, delayed development and others are elucidated in Table 3.

**Table 3:** Presenting features of children with spastic CP and associated abnormalities

Case no.	Name	Sex	Micro-cephaly	Associated abnormality with cerebral palsy		Other abnormalities
				Mental retardation	Developmental milestones	
1	KVM	F	+	−	Not attained any	Partial corpus callosal agenesis
2	CAK	M	+	+	Attained	Seizure disorder, B/L SNHL
3	KVD	M	−	−	Delayed	Birth asphyxia, hyperbilirubinemia
4	ARG	M	+	−	Global delay	Seizure disorder
5	JRK	M	−	+	Global delay	Corpus callosal agenesis, SNHL
7	BDC	M	+	+	Global delay	Seizure disorder, primary Optic atrophy
8	RGT	M	−	+	Attained	Typical febrile seizure
10	SBK	M	+	+	Attained	No seizure disorder
13	ASN	F	−	−	Attained	Cerebral atrophy, SNHL
14	NNM	F	−	−	Delayed	Frontal lobe atrophy

SNHL implies sensorineural hearing loss “+” implies present “−” implies absent

The above mentioned associated disorders found along with spastic CP are supported by CT and MRI investigations and the diagnosis was confirmed with the help of pediatrician’s opinion. The peculiar neurophysiological findings in these 10 cases have been represented in Table 4.

As far as the BAEP recordings are concerned, it is observed that average BAEP threshold for control group was 32.00 ±4.06 dB for left ear and 34.29 ± 5.02 dB for right ear whereas in patient group it was estimated as 44.67 ± 24.75 dB for left ear and 53.33 ± 28.45 dB for right ear.

**Table 4:** Peculiarities in neurophysiological findings of the above 10 cases of CP

Case no.	Name of subject	VEP abnormality		BAEP threshold	
				Left ear	Right ear
1	KVM	↓ P2 amplitude in LE	↑ Latency in RE	30 dB	40 dB
2	CAK	↓ P2 amplitude in LE	↑ Latency in RE	40 dB	60 dB
3	KVD	↓ P2 amplitude in LE	↑ Latency B/L	30 dB	40 dB
4	ARG	↓ P2 amplitude B/L	↑ Latency in RE	30 dB	80 dB
5	JRK	Markedly↓ amp. B/L	↑ Latency in RE	50 dB	40 dB
7	BDC	Markedly↓ P2 amplitude	↑ Latency B/L	30 dB	40 dB
8	RGT	Markedly ↓ P2 amplitude B/L	↑ Latency in RE	30 dB	30 dB
10	SBK	P2 amplitude WNL B/L	↑ Latency B/L	30 dB	30 dB
13	ASN	Markedly ↓ P2 amplitude B/L	↑ Latency in RE	50 dB	100 dB
14	NNM	↓ P2 amplitude in LE	↑ Latency in RE	30 dB	30 dB

The BAEP parameters of all the 15 children with CP are shown in Table 5. and mean latencies and amplitudes of BAEP waveforms are depicted in Table 6. Striking BAEP abnormalities in CP patients include prolongation of absolute latency of wave V, interpeak latencies of III–V and lowered V–I ratio as compared to the controls. The difference of means of all the BAEP parameters was statistically significant between the two groups (*P*<0.05). Abnormal VEPs and BAEPs in children with spastic cerebral palsy demonstrated a significant correlation with the presence of global developmental delay.

**Table 5:** BAEP parameters of the children with CP

Case no.	Absolute latencies left ear			Interpeak latencies left ear			V: I ratio LE	Absolute latencies right ear			Interpeak latencies right ear			V: I ratio RE
	I	III	V	I-III	III-V	I-V		I	III	V	I-III	III-V	I-V	
1.	3.77	5.85	7.23	2.08	1.38	3.46	1.27	4.21	5.48	7.63	1.27	2.15	3.42	3.55
2.	3.1	5.4	6.98	2.3	1.58	3.88	2.11	3.67	5.08	7.27	1.41	2.19	3.6	2.12
3.	3.65	5.96	7.65	2.31	1.69	4	6.17	3.52	5.69	7.42	2.17	1.73	3.9	2.57
4.	3.1	5.08	7.13	1.98	2.05	4.03	1.02	2.38	4.85	6.88	2.47	2.03	4.5	11.7
5	2.19	3.9	6.1	1.71	2.2	3.91	1.1	2.71	4.54	6.21	1.83	1.67	3.5	2.48
6	2.63	5	6.79	2.37	1.79	4.16	0.97	3.73	5.4	7.63	1.67	2.23	3.9	1.84
7.	3.13	5.31	7.4	2.18	2.09	4.27	2.93	3.13	5.13	7.15	2	2.02	4.02	5.26
8.	3.02	4.71	6.58	1.69	1.87	3.56	2.49	3.19	5.73	7.71	2.54	1.98	4.52	3.81
9.	1.8	3.54	5.87	1.74	2.33	4.07	1.5	1.65	3.96	5.98	2.31	2.02	4.33	1.25
10	3.63	5.77	7.79	2.14	2.02	4.16	5.68	3.35	5.52	7.21	2.17	1.69	3.86	2.25
11	1.73	4.29	6.15	2.56	1.86	4.42	1.69	3.35	4.69	6.04	1.34	1.35	2.69	4.13
12	3.42	5.4	7.04	1.98	1.64	3.62	3.3	2.54	5.17	6.92	2.63	1.75	4.38	1.03
13	2.29	5.04	6.02	2.75	0.98	3.73	1.28	1.48	3.81	5.65	2.33	1.84	4.17	0.97
14	2.92	4.54	5.96	1.62	1.42	3.04	2.15	2.5	4.96	6.92	2.46	1.96	4.42	2.93
15	3.08	5.88	7.77	2.8	1.89	4.69	6.89	2.94	5.96	7.92	3.02	1.96	4.98	7.25

**Table 6:** Mean Latencies and Amplitudes of BAEP Recordings

Groups	Absolute latencies left ear			Interpeak latencies left ear			V: I Ratio Lt Ear	Absolute latencies right ear			Interpeak latencies right ear			V: I ratio RE
	I	III	V	I-III	III-V	I-V		I	III	V	I-III	III-V	I-V	
Patient group (mean±SD) No. = 15	2.90 ± 0.65	5.04 ± 0.73	6.83 ± 0.68	2.15±0.37	1.79 ± 0.35	3.93 ± 0.41	2.70 ± 1.97	2.96±0.76	5.06±0.62	6.97±0.70	2.11±0.51	1.90±0.23	4.01±0.56	3.54±2.81
Control group (mean±SD) No. = 35	3.03±0.50	5.32±0.51	6.07±0.48	2.55±1.67	1.75±0.27	4.04±0.45	5.50±4.75	3.21±0.65	5.53±0.55	7.42±0.54	2.32±0.58	1.90±0.37	4.21±0.66	6.14±6.19
Unpaired t test	*P*<0.05	*P*<0.05	*P*<0.05	*P*<0.05	*P*<0.05	*P*<0.05	*P*<0.05	*P*<0.05	*P*<0.05	*P*<0.05	*P*<0.05	*P*<0.05	*P*<0.05	*P*<0.05

## Discussion

**Figure 1:**
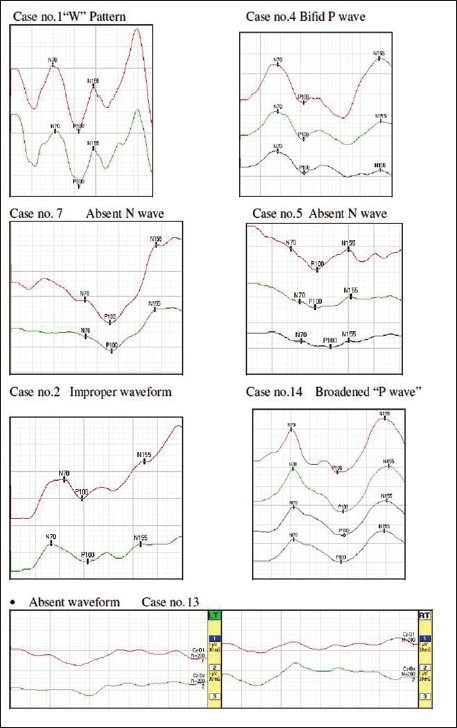
Variations in VEP Waveforms of children with CP

This article describes neurophysiological study using flash VEPs and BAEPs in children with CP. It was observed that those children who had other neurological symptoms associated with CP showed extensive deviation from the regular pattern of waveforms obtained in VEP investigations some of which are depicted in the Figure 1. This is in consonance with the notion put forward by previous workers that children with CP have abnormalities of the visual sensory and motor pathways at rates exceeding those detected in neurologically normal children.[11–17]

In a recent neurophysiologic study[9] on CP patients, increased latencies of VEPs were reported to occur more frequently with alterations in the optic radiation supported by MRI findings. Similar results are obtained in our study in those patients where their MRI findings reveal cerebral disorders such as corpus callosal agenesis, frontal lobe atrophy, cerebral atrophic changes etc.

It has been documented in the past[18] that microcephaly leads to more than 40% reduction in VEP amplitude. The small head size might be a possible cause of reduced VEP amplitudes found in microcephalic children of the present study.

The abnormal flash VEP findings of our study in cases of CP with developmental delay are in agreement with a most recent study[10] in children with bilateral spastic CP in which they have demonstrated significant correlation of VEP with moderate to severe developmental delay.

Nowadays the neurophysiologic techniques especially VEPs are able to demonstrate the brain plasticity in children with cerebral palsy. The mechanisms of plasticity have been postulated as a change in the balance of excitation and inhibition; a long-term potentiation or long-term depression; a change in neuronal membrane excitability; the anatomical changes-formation of new axon terminals and new synapses.[2]

It has been suggested that children with CP have a remarkable ability to recover from early brain injures. The utility of the neurophysiologic investigations like VEPs in the determination of brain reorganization and repair in patients with cerebral palsy (CP) has been described in a study.[9] According to this, VEP can be a useful tool in the determination of brain plasticity in children with CP as it has been suggested that VEP latencies are valuable estimators of neuronal injury and even predictors of later intellectual performance.[19]

**Figure 2:**
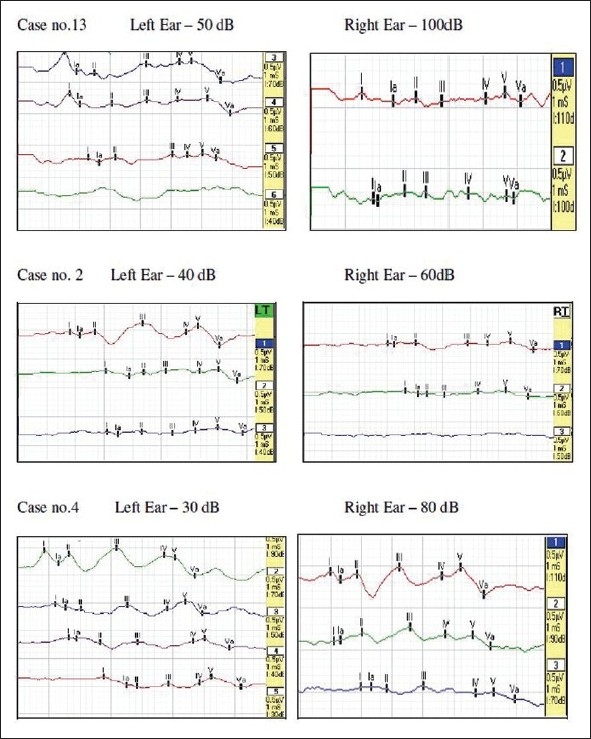
BAEP Threshold in waveforms of children with CP

High frequency of abnormal BAEP has been reported in children with spastic cerebral palsy. The BAEP abnormality has been significantly related by previous researchers to term delivery, perinatal etiology of cerebral palsy, visual abnormality, inability to walk independently and many more aspects. In a retrospective study on 75 children with spastic cerebral palsy, 22.7% had abnormal BAEP.[20] In our study profound sensorineural hearing loss (SNHL) was observed in case no.13 with CP along with cerebral atrophy. Bilateral mild to moderate SNHL was evident in a case no. 2 with CP along with MR, microcephaly and seizure disorder [Figure 2]. Moderate SNHL was also seen in right ear of case no.4 who had Eustachian tube defect in the same ear and in case no.5 who had raised threshold in left ear.

## Conclusion

The differences in VEPs and BAEPs were determined between CP children and healthy children. It is concluded that VEPs of the patient group revealed a marked delay in the latency as well as a reduction in the amplitude apart from certain variations in the waveforms. BAEP abnormalities in CP patients include prolongation of absolute latency of wave V, interpeak latencies of III–V and lowered V–I ratio as compared to the controls. The average threshold for hearing also greatly increased in the patient group as compared to controls. The abnormalities found are probably linked to the neurological deficits present in cases of cerebral palsy although the exact etiology remains to be unleashed. Further to conclude, since VEPs and BAEPs are non-invasive neurophysiological investigations; they can serve as important tools in the diagnostic work up of spastic cerebral palsy.
